# High-efficiency editing in hematopoietic stem cells and the HUDEP-2 cell line based on *in vitro* mRNA synthesis

**DOI:** 10.3389/fgeed.2023.1141618

**Published:** 2023-03-08

**Authors:** Nikoletta Y. Papaioannou, Petros Patsali, Basma Naiisseh, Panayiota L. Papasavva, Lola Koniali, Ryo Kurita, Yukio Nakamura, Soteroula Christou, Maria Sitarou, Claudio Mussolino, Toni Cathomen, Marina Kleanthous, Carsten W. Lederer

**Affiliations:** ^1^ Department of Molecular Genetics Thalassemia, The Cyprus Institute of Neurology and Genetics, Nicosia, Cyprus; ^2^ Research and Development Department, Central Blood Institute, Blood Service Headquarters Japanese Red Cross Society, Tokyo, Japan; ^3^ Cell Engineering Division, RIKEN BioResource Research Center, Tsukuba, Japan; ^4^ Thalassaemia Centre, State Health Services Organisation of Cyprus, Nicosia, Cyprus; ^5^ Thalassaemia Centre, State Health Services Organisation of Cyprus, Larnaca, Cyprus; ^6^ Institute for Transfusion Medicine and Gene Therapy, Medical Center—University of Freiburg, Freiburg, Germany; ^7^ Center for Chronic Immunodeficiency (CCI), Faculty of Medicine, University of Freiburg, Freiburg, Germany

**Keywords:** in vitro transcription, mRNA, genome editing, hematopoietic, CRISPR/Cas, base editor

## Abstract

**Introduction:** Genome editing tools, such as CRISPR/Cas, TALE nucleases and, more recently, double-strand-break-independent editors, have been successfully used for gene therapy and reverse genetics. Among various challenges in the field, tolerable and efficient delivery of editors to target cells and sites, as well as independence from commercially available tools for flexibility and fast adoption of new editing technology are the most pressing. For many hematopoietic research applications, primary CD34^+^ cells and the human umbilical cord-derived progenitor erythroid 2 (HUDEP-2) cell line are highly informative substrates and readily accessible for *in vitro* manipulation. Moreover, *ex vivo* editing of CD34^+^ cells has immediate therapeutic relevance. Both cell types are sensitive to standard transfection procedures and reagents, such as lipofection with plasmid DNA, calling for more suitable methodology in order to achieve high efficiency and tolerability of editing with editors of choice. These challenges can be addressed by RNA delivery, either as a mixture of guide RNA and mRNA for CRISRP/Cas-based systems or as a mixture of mRNAs for TALENs. Compared to ribonucleoproteins or proteins, RNA as vector creates flexibility by removing dependence on commercial availability or laborious in-house preparations of novel editor proteins. Compared to DNA, RNA is less toxic and by obviating nuclear transcription and export of mRNA offers faster kinetics and higher editing efficiencies.

**Methods:** Here, we detail an *in vitro* transcription protocol based on plasmid DNA templates with the addition of Anti-Reverse Cap Analog (ARCA) using T7 RNA polymerase, and poly (A) tailing using poly (A) polymerase, combined with nucleofection of HUDEP-2 and patient-derived CD34^+^ cells. Our protocol for RNA-based delivery employs widely available reagents and equipment and can easily be adopted for universal *in vitro* delivery of genome editing tools.

**Results and Discussion:** Drawing on a common use case, we employ the protocol to target a β-globin mutation and to reactivate γ-globin expression as two potential therapies for β-hemoglobinopathies, followed by erythroid differentiation and functional analyses. Our protocol allows high editing efficiencies and unimpaired cell viability and differentiation, with scalability, suitability for functional assessment of editing outcomes and high flexibility in the application to different editors.

## 1 Introduction

In the past two decades, *ex vivo* genome editing in hematopoietic stem and progenitor cells (HSPCs) has shown promising results, rendering it an ideal therapy approach for many hematological monogenic diseases (Koniali et al., 2021), including even clinical application for the β-hemoglobinopathies, β-thalassemia and sickle cell disease (Frangoul et al., 2021). Progress and relevance of HSPC-based research in advanced therapies and reverse genetics increasingly rely on a fast-expanding arsenal of editing tools (Canver et al., 2015; Patsali et al., 2019b; Zeng et al., 2020; Tucci et al., 2022), so that efficiency, tolerability and versatility of delivery methods are paramount.

The currently most popular editing platforms are the now ubiquitously applied clustered regularly interspaced short palindromic repeats (CRISPR)/CRISPR-associated (Cas) protein system, the transcription activator-like effector nucleases (TALENs) and the zinc finger nucleases (ZFNs), all of which in their original form introduce site-specific double-strand breaks (DSBs) as the basis for DNA sequence alterations. The DSBs are mostly resolved by two major DNA-repair mechanisms, the non-homologous end joining (NHEJ) mechanism, resulting in indels, or the precise homology-directed repair (HDR) mechanism in the presence of an HDR donor (Joung and Sander, 2013; Jiang and Doudna, 2017). ZNFs, as the oldest of the three platforms, are based on interdependent transcription factor binding domains (Kim et al., 1996; Urnov et al., 2005). By contrast, TALENs are based on a nucleotide-specific amino-acid code of repeat variable di-residues (RVDs), repurposing the dimeric FokI nuclease also employed in ZFNs (Boch et al., 2009; Moscou and Bogdanove, 2009; Mussolino et al., 2011). Finally, the CRISPR/Cas ribonucleoprotein (RNP) platform has enabled a marked acceleration and expansion of the field of DNA editing since its adaptation for use in eukaryotic cells in 2012 (Jinek et al., 2012). Inarguably, with target recognition based on complementarity of the DNA target to the CRISPR guide RNA (gRNA) and owing to a fast-growing variety of short protospacer-adjacent motif (PAM) sequences hardcoded in the Cas enzyme of choice (Walton et al., 2017; Gasiunas et al., 2020), CRISPR/Cas is presently the go-to platform for most DSB-based DNA editing applications to new targets. However, all DSB-based editing holds inherent risk of genotoxicity, so that several studies demonstrated that creation of DSBs through CRISPR/Cas9 can lead to apoptosis by induction of p53-mediated DNA damage response (Haapaniemi et al., 2018; Ihry et al., 2018), to undesired genomic alterations (large deletions, translocations) (Kosicki et al., 2018) and even chromothripsis (Leibowitz et al., 2021). One means of addressing safety concerns around genome editing is the development of methods to evaluate the safety of CRISPR-based therapies. For example, a recent study by Cromer et al. demonstrated a successful next-generation sequencing (NGS) workflow able to identify low-frequency events in HSPCs and showed that RNP delivery of high-fidelity Cas9 can prevent tumorigenic variants (Cromer et al., 2022). An alternative means of increasing safety is DNA editing without DSB induction.

Most recently, two pioneering CRISPR/Cas-based DSB-independent platforms for genome editing have been developed, which compared to DSB-dependent tools have lowered risk of enriching apoptosis-deficient cell populations and of recombination and indel events (Antoniou et al., 2021). Base editors (BEs) and prime editors (PEs) both aim to minimize DSB events by introducing single-stranded edits and employing nickase activity and local suppression of mismatch repair to encourage efficient DSB-independent double-strand editing. BEs employ chemical modification to catalyze base transition events and presently achieve outstanding editing efficiencies, whereas the larger prime editors (PEs) employ reverse transcription to synthesize a repair template *in situ* and have higher target and editing versatility. The well-established BEs comprise a nickase Cas9 fused to a deaminase enzyme (Rees and Liu, 2017) and exist in two types, the cytosine base editors (CBEs), which are composed of a cytidine deaminase and are able to convert a cytidine (C) into a thymidine (T) base (Komor et al., 2016), and the adenine base editors (ABE), which are composed of an adenine deaminase and are able to convert an adenine (A) into a guanine (G) base (Gaudelli et al., 2017). In a rapidly progressing expansion of our arsenal of editors, PEs have most recently become the basis for DSB-independent large targeted insertions (Yarnall et al., 2022), and novel glycosylase-based BEs even permit catalysis of C-to-G transversion events (Cao et al., 2022; Sun et al., 2022).

Despite such progress in the development of editing tools, choosing the optimal, i.e., the most affordable, tolerable and effective, delivery method still remains difficult, as different concerns must be considered, including host, target tissue and cell type, route of delivery and physical barriers (Khalil, 2020). Of particular therapeutic interest are HSPCs, which pose a particular challenge for the delivery of genome editing tools, since they are mostly quiescent cells and hard to transfect with plasmid DNA delivered either by lipid-based transfection or by nucleofection (Morgan et al., 2018). As preclinical progress is being made for *in vivo* delivery (Banskota et al., 2022; Eisenstein et al., 2022; Li et al., 2022), *ex vivo* electroporation is the clinically applied delivery method of choice for HSPCs (Frangoul et al., 2020), which optimized and applied at laboratory scale as nucleofection may reach exceptional efficiencies and tolerability for HSPCs (Patsali et al., 2019b; Laustsen and Bak, 2019). Nucleofection is possible for DNA, RNA, protein, and RNP cargo. Of these, plasmid DNA despite being most affordable and readily accessible, comes with a few shortcomings particularly in the sensitive HSPCs, such as low editing efficiencies, toxicity, persistence and the risk of insertional mutagenesis. By contrast, protein or RNP delivery allow high editing efficiencies and more transient delivery, but suffer from high cost or laborious in-house production of commercially unavailable editor proteins (Glass et al., 2018). Therefore, mRNA-based delivery of editor protein components for ZFNs, TALENs, and CRISPR/Cas, is a powerful alternative for protein and RNP-based editor platforms, as it is fast, practical and enables high editing efficiencies (Thuille et al., 2020). Compared to proteins or RNPs, RNA as delivery vehicle creates flexibility by removing dependence on commercial or in-house protein production. Compared to DNA, RNA as vector is less toxic, more transient and offers faster kinetics and higher editing efficiencies by obviating nuclear transcription and export of mRNA (Kwon et al., 2018).

Messenger RNA synthesis is achieved by using a bacteriophage RNA polymerase for *in vitro* transcription from a DNA template harboring corresponding transcriptional control elements. The most frequently used system is that of the T7 phage of *E. coli* (Beckert and Masquida, 2011), which reliably supports the full-length transcription of RNA spanning several kb. Importantly, most eukaryotic mRNAs carry a 7-methyl guanosine (m7G) cap at the 5′ end and a poly(A) tail at the 3’ end as essential components for efficient translation (Henderson et al., 2021). To mimic natural mRNAs and achieve high levels of translation, the synthetic RNA is therefore capped by co-transcriptional addition of an Anti-Reverse Cap Analog (ARCA) using the T7 RNA polymerase (Jemielity et al., 2003), while a poly(A) tail is added by inclusion of a poly(A) polymerase (Jalkanen et al., 2014; Henderson et al., 2021). Several studies have shown high editing efficiencies with the delivery of genome editing tools as mRNA, e.g., for TALENs (Quintana-Bustamante et al., 2019) or in combination with synthetic gRNA, for CRISPR/Cas9 (Newby et al., 2021) and BEs (Gaudelli et al., 2020), routinely achieving 70%–80% editing efficiency and resulting in low cytotoxicity (Bjurström et al., 2016).

Here, we present a detailed highly efficient *in vitro* transcription protocol based on the NEB HiScribe™ T7 ARCA mRNA kit with tailing, for production of editors as mRNA from plasmid DNA, and the delivery of these tools in hematopoietic cells, including the human umbilical cord-blood derived erythroid progenitor (HUDEP-2) cell line (Kurita et al., 2013) and patient-derived CD34^+^ HPSCs, based on nucleofection. To show general applicability, the protocol is used here to demonstrate suitability for both RNP- and protein-based editing platforms, first on BEs, because of their safety, efficiency and ease of application, and second on TALENs, because of their small size, their versatility, their being representative of DSB- and FokI-based editing (Mok et al., 2020; 2022), and their uncomplicated licensing for commercial use (Farooqui, 2019; Ledford, 2022). A well-understood system for the evaluation of new methodology is the β-globin disorders, β-thalassemia and sickle disease (Lederer and Kleanthous, 2015), which can both be corrected either by inducing γ-globin as a therapeutic β-globin ortholog and anti-sickling agent (Fanis et al., 2019; Wu et al., 2019; Ravi et al., 2022) or by addressing the primary mutation (Patsali et al., 2019a; Patsali et al., 2019b). Applying, i) gRNA/mRNA-based delivery for BE technology to induce γ-globin and ii) mRNA-based delivery for TALEN technology to disrupt an intronic β-globin mutation, our analyses indicate that the current protocol allows high editing efficiencies and unimpaired erythroid differentiation, correction of disease parameters and functional analyses.

## 2 Materials and methods

### 2.1 Cell culturing

The HUDEP-2 cell line and patient-derived CD34^+^ HPSCs were used. Peripheral blood samples from patients were acquired by fully informed consent (Cyprus National Bioethics Committee license number ΕΕΒΚ/ΕΠ/2018/52) and CD34^+^ cells isolated by magnetic-activated cell sorting (Miltenyi Biotec, Bergisch Gladbach, Germany). Both cell types were kept in expansion for approximately 6–7 days and were then subjected to erythroid differentiation, up to 9 days. The CD34^+^ isolation procedure, expansion and differentiation conditions for both HUDEP-2 and CD34^+^ cells, and the medium used for both cell types were as described elsewhere (Papasavva et al., 2021).

### 2.2 Plasmid purification and verification

All plasmids used were either purchased from Addgene (Watertown, MA, United States), specifically pCMV_BE4max (Addgene ID: 112093), or previously published (Patsali et al., 2019b), and retargeting of BEs was achieved by co-transfection with alternative synthetic gRNAs. Purchased plasmids were received as bacterial stabs, which were purified and verified. Briefly, bacteria were spread in an agar plate following overnight (O/N) incubation at 37°C. A single colony was isolated and added in Luria Broth (LB) (Invitrogen, Carlsbad, CA, United States), Supplemented with antibiotic (ampicillin or kanamycin), and was incubated O/N at 37°C. The next day, the plasmids were purified with the Nucleobond Xtra Midi Endotoxin-free plasmid DNA purification kit (Macherey-Nagel GmbH & Co. KG, Düren, Germany) following the manufacturer’s instructions. Approximately 1 μg of each plasmid was used to verify identities by informative restriction digests and comparison with the corresponding plasmid map.

### 2.3 *In vitro* transcription

*In vitro* transcription was performed using the HiScribe™ T7 ARCA mRNA kit (with tailing) (#E2060S) (New England Biolabs, Ipswich, MA, United States), with great care to keep all equipment and reagents sterile and RNase-free. As of this writing, reagent prices per 2 × 10^5^ cells of starting material amount to approximately €20 for BEs and €36–48 for TALENs (depending on the amount of mRNA used), including gRNA for BEs and *in vitro* transcription reagents (steps included in Sections 2.3.3–Section 2.3.7), but excluding consumable costs for DNA purification, nucleofection, culture and subsequent analyses. Additional costs can be reduced, e.g., by re-use of nucleofection cuvettes and alternative electroporation buffer (Bak et al., 2018).

#### 2.3.1 DNA template preparation

Efficient *in vitro* transcription using this protocol requires a T7 promoter on a linearized plasmid DNA (or as part of a PCR product). Here, we used approximately 6–10 μg of plasmid DNA for linearization by restriction enzyme digestion downstream of the open reading frame (ORF), as a template for mRNA synthesis. For all constructs, the *PmeI* enzyme (10 Units/μL) was used in 1× CutSmart Buffer with DNA template and water up to 50 μL total reaction volume. The reaction was incubated O/N at 37°C. Complete linearization is critical for successful mRNA synthesis and was confirmed by loading a small quantity of the sample on a 0.8% agarose gel along with an undigested DNA plasmid.

#### 2.3.2 DNA purification and quantification

The digested DNA template must be highly purified, which can be accomplished by any regular column-based kit or by phenol:chloroform and ethanol extraction. From this step onwards, using RNase-free reagents and, where possible, equipment and plasticware used exclusively for RNA work, will greatly contribute to high RNA yield and quality. For column-based purification we applied the QIAquick PCR purification kit (Qiagen, Hilden, Germany) according to the manufacturer’s instructions with the final elution step in only 30 μL RNase-free water. For phenol:chloroform extraction, 350 μL of RNase-free water was added to the DNA, followed by an equal volume (400 μL) of acid-phenol: chloroform (Sigma-Aldrich, Munich, Germany). After a brief vortex and centrifugation for 5 min at maximum speed (≥17,000 × g) and at room temperature (RT), the upper aqueous phase was transferred to a new tube and an equal volume of chloroform (Sigma-Aldrich, Munich, Germany) was added. After renewed phase separation by centrifugation, the aqueous phase is again transferred in a fresh sterile tube, before ethanol (EtOH) precipitation by addition of 1.2 μL RNA-grade glycogen (Invitrogen, Massachusetts, United States), 0.1 volume 3 M sodium acetate and 2.5 volumes 100% ice-cold EtOH, by a brief vortex and by O/N incubation at −20°C. The next day, the sample was centrifuged for 30 min at maximum speed and 4°C, and the pellet washed in 500 μL ice-cold 75% EtOH by centrifugation for 5 min at 7,500 × g and 4°C. The air-dried pellet was dissolved in 30 μL RNase-free water. A nanodrop spectrophotometer was used to determine DNA concentration and purity. A concentration of 150–200 ng/μL was considered ideal for the following steps, and DNA was considered sufficiently pure with an A_260_/A_280_ ratio >1.8 and an A_260_/A_230_ ratio in the range of 2.0–2.2.

#### 2.3.3 Standard mRNA synthesis

The reaction was assembled at RT as follows: For BE production, a 20-μL reaction were set up by adding to nuclease-free water, 10 μL 2× ARCA/NTP mix, 1 μg template DNA, and 2 μL T7 RNA polymerase mix. For TALEN production, the same order was followed, but the reaction was scaled up to 30 μL based on 1.5 μg of purified DNA and 3 μL of T7 RNA polymerase mix. Reactions were incubated at 37°C for 45 min, with possibility of subsequent storage of the capped RNA at −20°C for several days.

#### 2.3.4 DNase treatment

The capped RNA was treated to remove any DNA residuals by adding 1/10 of initial reaction volume of DNase I, followed by 37°C incubation, of the resulting 22-μL reaction for 30 min or of the resulting 33-μL reaction for 40 min.

#### 2.3.5 Poly(A) tailing

The tailing reaction was set up at RT in 50 μL final volume as follows: to 20 μL nuclease-free water were added 20 μL of the DNase-treated capped RNA, 5 μL of 10× Poly(A) Polymerase reaction buffer and 5 μL Poly(A) polymerase. For the TALENs, the reaction was scaled up to 100 μL final volume. The reaction was incubated at 37°C for 30 min.

#### 2.3.6 mRNA purification

The final modified mRNA was purified using LiCl precipitation. Briefly, 25 and 50 μL LiCl were added to the 50 and 100 μL tailing reaction, respectively, were vortexed briefly, incubated for 1 h at −20°C, and centrifuged at 4°C for 15 min at maximum speed. The resulting pellet was washed with 500 μL of ice-cold 70% EtOH followed by centrifugation at 4°C for 10 min at maximum speed. After the pellet was air-dried, it was resuspended with RNase-free water (40 μL for BEs or 11 μL for TALENs). The final mRNA was heated at 65°C for 5 min and after evaluating concentration and purity (step 2.3.7.) was aliquoted according to the requirements of the experiment, and stored at −80°C.

#### 2.3.7 Evaluation of mRNA product

Messenger RNA purity and quantity was evaluated by NanoDrop spectrophotometry of a 1:10 dilution. For mRNA, an A_260_/A_280_ ratio of >2 and an A_260_/A_230_ ratio between 2.0–2.2 indicated acceptable purity for downstream application. Integrity of mRNA was analyzed on a 1% TBE agarose gel along with a ssRNA ladder (New England Biolabs, Ipswich, MA, United States), using RNase-free equipment and reagents. After samples were heat-treated at 65°C for 5 min, they were run with the gel tank on ice, at 90 V/11 cm electrode distance for approximately 30 min before visualization on a Vilber FUSION FX7 bioimager (Vilber Lourmat Sté, Collégien, France).

### 2.4 Nucleofection

Cells were nucleofected with a 4D-Nucleofector^™^ instrument using the P3 Primary Cell 4D-Nucleofector X Kit (both Lonza, Basel, Switzerland) and applying the CA-137 program. Approximately 2–3 × 10^5^ cells were used for each experiment. For the nucleofection of BEs a mixture of *in vitro* transcribed BE mRNA and a chemically modified gRNA (Synthego, Redwood City, CA, United States) (for gRNA sequences see Supplementary Table S1) was made using a 2:1 ratio (2 μg gRNA:1 μg BE mRNA) as the optimal ratio based on efficiency, toxicity and cost. For the nucleofection of TALENs, a mixture of mRNA encoding left and right TALEN monomer was made (for RVD sequences see Supplementary Table S2) using a 1:1 ratio with 2 μg or 1.5 μg of each construct. The cells were centrifuged at 250 ×g for 5 min and the pellet was resuspended in 20 μL of nucleofection buffer for addition to the corresponding reagent mixture. The resulting final reaction mix was loaded in 16-well Nucleocuvette^™^ strips (Lonza, Basel, Switzerland) and transfected in the Nucleofector. Finally, cells were placed in expansion medium in a 24-well plate (SPL Life Sciences, Gyeonggi-do, South Korea). For TALENs, cells were cultured at hypothermic (32°C) conditions for the first 16–24 h post-nucleofection.

### 2.5 Assessment of DNA editing

#### 2.5.1 Genomic DNA purification and sequencing

Five to seven days post-nucleofection, approximately 3 × 10^5^ cells were collected for DNA analysis. The genomic DNA was extracted from cells using the QIAmp DNA Blood Mini Kit (Qiagen, Hilden, Germany) according to the manufacturer’s instructions, and was amplified by polymerase chain reaction (PCR) using Q5^®^ Hot Start High-Fidelity DNA Polymerase (New England Biolabs, Ipswich, MA, United States). The PCR product was purified using the QIAquick^®^ PCR Purification Kit (Qiagen Hilden, Germany) and was then processed for Sanger sequencing (for primer sequences see Supplementary Table S3) as the basis for the assessment of DNA editing. For cycle sequencing, the BigDye^™^ Terminator v1.1 Cycler Sequencing Kit (Applied Biosystems, Waltham, MA, United States) was used according to the manufacturer’s instructions.

#### 2.5.2 Deconvolution of sequence traces

For DNA-level evaluation of base editing, the EditR software (Kluesner et al., 2018) was used. Briefly, Sanger sequencing trace files and gRNA sequence were uploaded to display the area percentage for A|C|G|T base signals at each position of the gRNA and to score deviation from the original sequence as editing. For the evaluation of DSB-based editing, we adopted procedures for the Inference of CRISPR Edits (ICE) software (Conant et al., 2022), in which Sanger sequencing trace files of a control and of edited samples were uploaded, together with an indication of the approximate DSB site, to infer a percentage contribution of different indel events after editing.

### 2.6 Whole-cell lysate extraction and immunoblotting

Whole-cell lysate for protein analyses was extracted from cells using 20 μL of radioimmunoprecipitation assay (RIPA) lysis buffer (20 mM Tris-HCl pH 7.4, 150 mM NaCl, 1% Nonidet P-40, 0.1% sodium dodecyl sulfate, 0.5% sodium deoxycholate, 5 mM EDTA) per 1 × 10^6^ cells, Supplemented with 100× protease inhibitors (Roche, Basel, Switzerland). After 20 min incubation on ice and centrifugation at 4°C for 10 min at 16,000 ×g, the supernatant was collected and an equal volume of 2× Sample Buffer (5 mL 0.25 M Tris pH 6.8, 2 mL 10% SDS, 2.1 mL glycerol, 0.4 mL β-mercaptoethanol, 0.5 mL 0.1% bromophenol) was added. Proteins were separated by SDS-PAGE gel and were then transferred to a Nitrocellulose Parablot NCP membrane (Macherey-Nagel GmbH & Co. KG, Düren, Germany). After the membrane was blocked with 1% bovine serum albumin (BSA) (Roche, Basel, Switzerland), it was incubated O/N with primary antibody followed by a 1-h incubation with the corresponding horseradish-peroxidase-conjugated secondary antibody (for antibodies see Supplementary Table S4). The banding patterns were covered with chemiluminescence staining buffer (BioRad, Hercules, United States) and visualized with a Vilber FUSION FX7 imaging system (Vilber Lourmat Sté, Collégien, France). For quantification, the Evolution-Capt Edge software (Vilber Lourmat Sté, Collégien, France) was used, and all samples were normalized based on β-actin levels.

### 2.7 High-performance liquid chromatography (HPLC)

Reversed-phase (RP) HPLC was performed as detailed elsewhere (Loucari et al., 2018), but with cell lysis in 5 mM 1,4-dithiothreitol (DTT) instead of HPLC-grade distilled water.

## 3 Results

### 3.1 Production of functional mRNA

**FIGURE 1:**
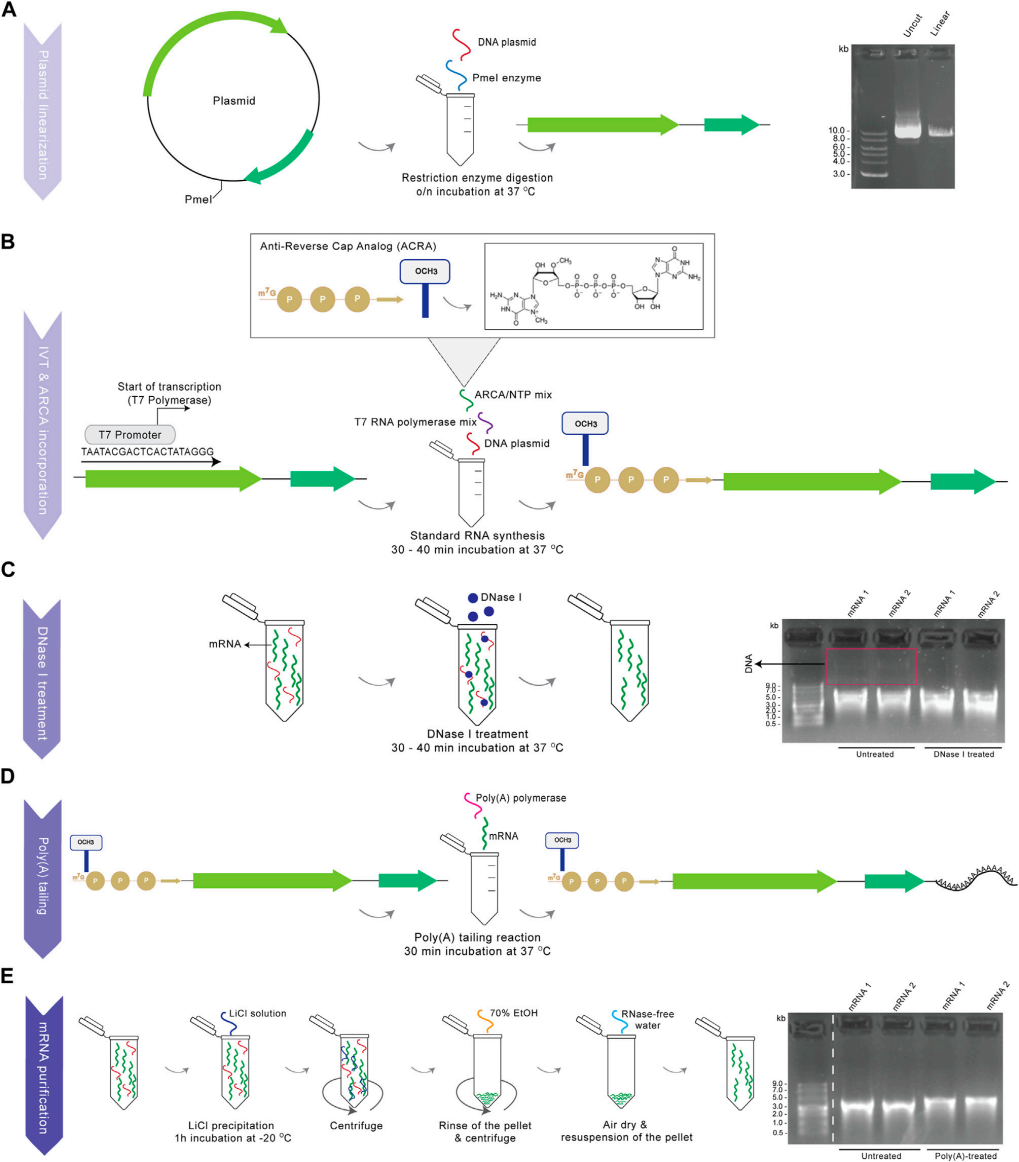
Production of functional mRNA. Schematic diagram showing all the steps for the current *in vitro* transcription protocol, with corresponding gel images for the example of two representative BE mRNA synthesis procedures (1,2) from the same plasmid. **(A)** Linearization of plasmid DNA by restriction enzyme digestion downstream of the ORF to create a template for mRNA synthesis. After step A, uncut and linearized plasmid was loaded on a 0.8% agarose gel, along with a 1 kb DNA ladder (gel image) **(B)** Synthesis of a capped mRNA by co-transcriptional incorporation of ARCA using T7 RNA polymerase. **(C)** DNase I treatment of the capped mRNA for the removal of DNA residuals. The mRNA product was loaded on a 1% agarose gel untreated (from step B) and DNase I treated (from step C) (gel image) **(D)** Tailing reaction using poly(A) polymerase for the addition of a poly(A) tail to the capped mRNA. **(E)** Purification of the final modified mRNA following LiCl precipitation. Finally, the capped RNA product was loaded on a 1% agarose gel untreated (from step D) and purified and poly(A)-treated (from step E), along with a ssRNA ladder (gel image). Dashed white lines indicate where images were simplified by removal of surplus lanes.

Procedures and reagents for mRNA synthesis are detailed in Materials and Methods to facilitate adoption of our procedures. Owing to chemical lability of RNA and the ubiquity of RNases, production and application of synthetic mRNA might be challenging (Wadhwa et al., 2020). This necessitates confirmation of integrity, length, quantity and purity of products, and great care in the avoidance of RNase contamination of reagents and equipment, every step of the way (Figure 1). A first key step of the procedure is complete restriction digestion of the DNA plasmid (Figure 1A), which guarantees mRNA products of defined length and sequence, and avoids otherwise abundant readthrough transcription products. Linearization is followed by T7-driven RNA transcription and co-transcriptional capping with ARCA, before DNase I treatment for removal of the template DNA (Figure 1C), which would otherwise interfere with downstream experiments. As a final step, poly(A) tailing is performed, which is readily detectible by gel separation as a size increase of poly(A)-tailed mRNA compared to untreated RNA and which for successful production shows the pure mRNA transcript as a clear, single band without lower–molecular-weight (MW) smear indicative of degradation, and without the presence of higher-MW residual plasmid DNA (Figures 1D, E).

### 3.2 Delivery of base editors as gRNA/mRNA for γ-globin induction in hematopoietic cells

Base editing technology was exploited to target γ-globin modifiers as potential therapy for β-hemoglobinopathies. Use of HUDEP-2 cells, with their minimal baseline γ-globin expression, facilitates such analyses (Papasavva et al., 2022), but owing to low transfection efficiency and high toxicity of plasmids in these sensitive cells, calls for *in vitro* transcribed mRNA in combination with commercial gRNA for delivery of BEs.

**FIGURE 2:**
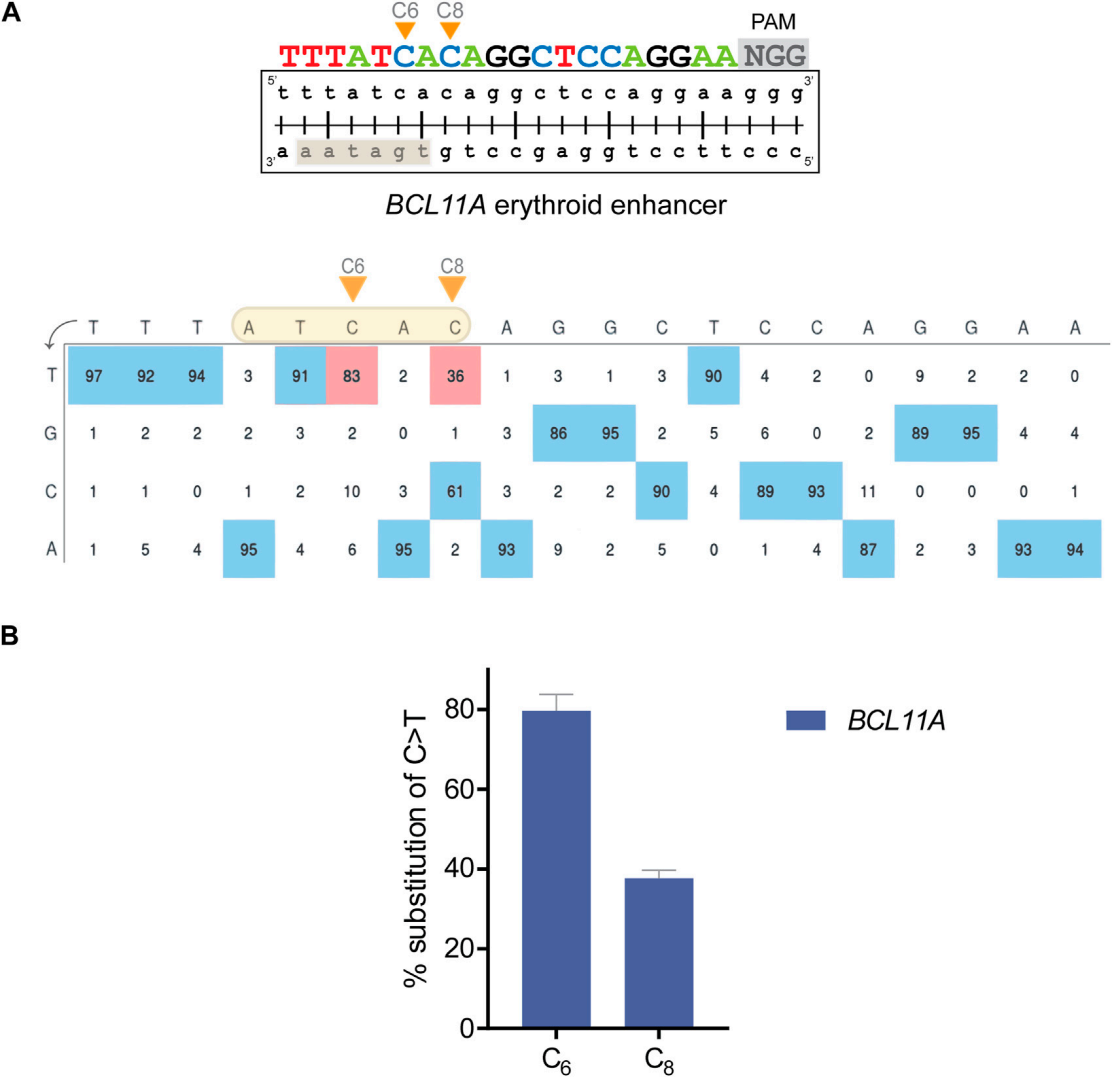
Editing efficiency with BE in HUDEP-2 cells. **(A)** (top) Schematic diagram illustrating the *BCL11A* target with the DNA-equivalent of the 20-nt gRNA and the 3-nt PAM sequence. The orange arrows show the C bases that were edited. The highlighted rectangle indicates the WGATAR binding motif in *BCL11A*. (bottom) Annotated EditR-generated plot illustrating the percent area of the signal for each base (A*|*C*|*G*|*T) at the corresponding gRNA position for edited HUDEP-2 cells. The highlighted shape shows the editing window, the orange arrows show the edited C bases and the red boxes display the exact percentage (%) of base substitution in the bulk cell population. **(B)** Chart showing the % base substitution of C>T after base editing. Each bar shows the editing of the corresponding C base along the gRNA. The data are plotted as mean ± s.d. (*n* = 3).

Literature supports that amelioration of β-hemoglobinopathies can be achieved by HbF-inducing mutations in regulatory sequences (Gallienne et al., 2012; Steinberg, 2020). Such mutations can often be created by single transition mutations and are usually reinforced, rather than reduced, in their effect by inadvertent on-target bystander edits, which altogether renders them ideal targets for γ-globin induction by base editing. To this end and using the BE4max cytosine BE (Koblan et al., 2018), we targeted the well-characterized γ-globin repressor *BCL11A*, so as to disrupt the *GATA1* binding motif within the +58 *BCL11A* erythroid enhancer (Figure 2A) (Zeng et al., 2020). To indicate the potential of our protocol for reverse genetics, we also applied the BE4max BE mRNA with the appropriate gRNAs to target the *HBG* promoter for the creation of −114 and −115 C>T mutations (Martyn et al., 2018; Zhang et al., 2020) and to target γ-globin repressor *KLF1* for the introduction of a p.Glu5Lys (C>T) mutation (Liu et al., 2014), as additional *cis* and *trans* factors of γ-globin expression, respectively (Supplementary Figure S1A). For the *HBG* promoter, specific point mutations result in hereditary persistence of fetal hemoglobin (HPFH), a benign condition characterized by elevated γ-globin expression. Furthermore, it was found that the −114 or −115 C>T mutations at the *HBG* promoter disrupt the binding site of the strong γ-globin repressor BCL11A (Martyn et al., 2018; Zhang et al., 2020). For *KLF1* as an indirect repressor of γ-globin, numerous point mutations have been associated with elevated adult γ-globin levels (Gallienne et al., 2012), warranting the functional analysis of any novel variants of unknown significance in the *KLF1* gene for their potential to induce γ-globin production and serve as a basis for therapy development.

Cells were nucleofected and cultured, before editing was assessed by deconvolution of mixed sequence traces using EditR (Kluesner et al., 2018) to infer the percentage substitution of each base along the gRNA (Figure 2A; Supplementary Figure S1A) and to evaluate editing across up to three independent biological replicates (Figure 2B). For the *BCL11A* erythroid enhancer, 2 C bases of the *GATA1* binding motif within the predicted editing window, specifically the C bases at positions 6 and 8 (referred to as “C6” and “C8”, respectively) were edited at high efficiency in the absence of bystander edits outside that window. The C>T editing frequencies were 79.7% ± 4.2% and 37.7% ± 2.1% C>T for C6 and C8, respectively (*n* = 3) (Figure 2B). For the *HBG* target and its suitably positioned editing window, C6 and C7 were edited 30.3% ± 7% and 31.3% ± 7.5% C>T, respectively, in the absence of bystander edits (*n* = 3). For the *KLF1* target, C3 and C5 were edited 49% and 83% C>T, respectively (*n* = 1). Importantly, *KLF1* C5 is located in the editing window and was the intended editing target to create a GAG>AAG (Glu > Lys) mutation, while base editing of C3 as an inadvertent bystander target resulted in a synonymous AAG>AAA (Lys > Lys) ORF mutation, maintaining the intended Glu5Lys codon change (Supplementary Figure S1B).

**FIGURE 3:**
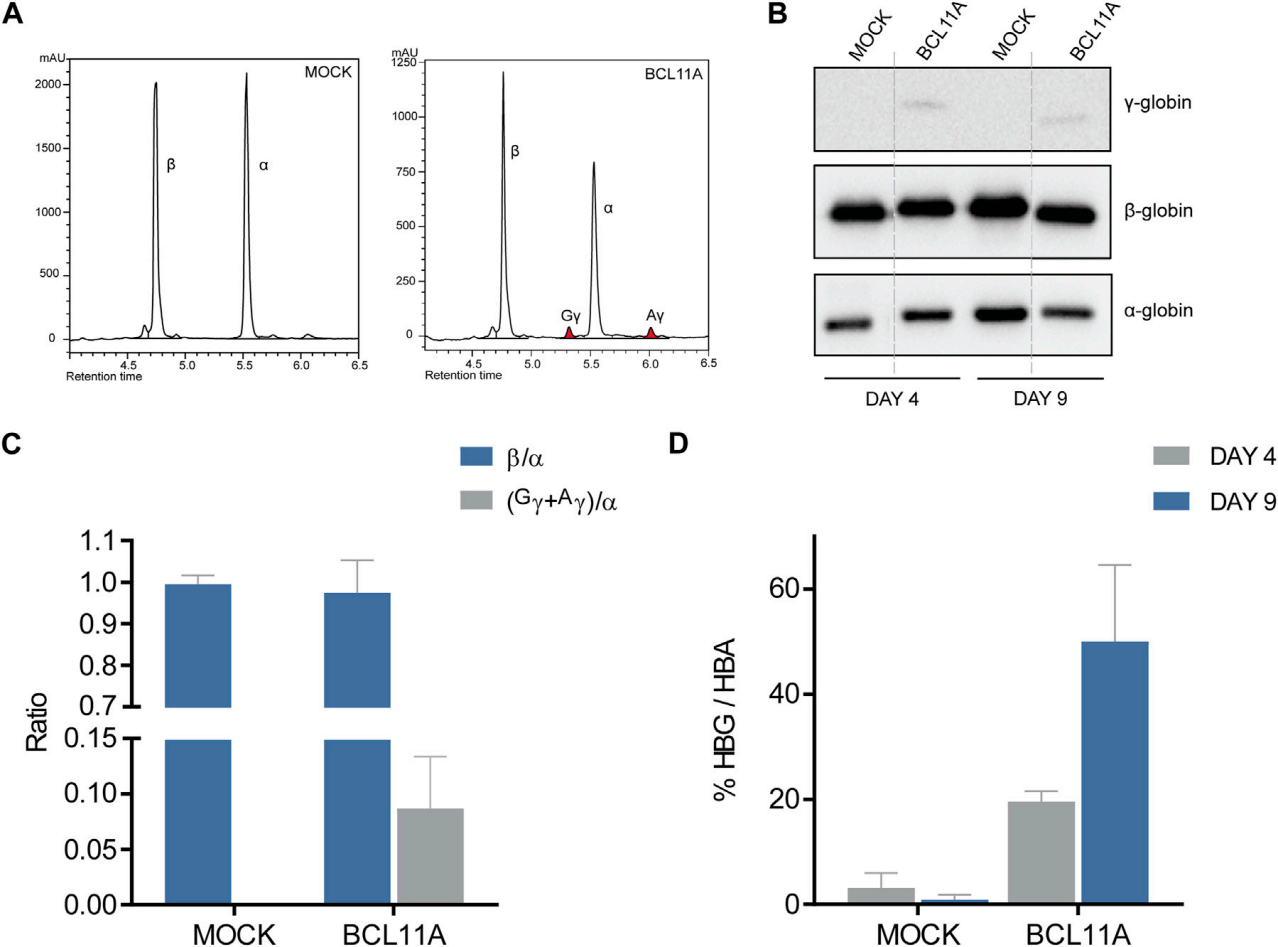
Functional analysis of BE-modified HUDEP-2 cells. **(A)** Chromatograms of HPLC analysis for the *BCL11A* and mock targeting at the last day of HUDEP-2 erythroid differentiation, with the peaks showing globin expression. The α-, β- and γ-globin peaks are labelled, and colored peaks indicate γ-globin induction. **(B)** Immunoblots of the edited HUDEP-2 cells on days 4 and 9 of erythroid differentiation, detecting the protein expression of α-, β- and γ-globin. Dashed lines indicate where images were simplified by removal of surplus lanes (see Supplementary Figure 2 for the full image). **(C)** Quantification of HPLC analysis in a chart showing the globin ratios of β/α and (^G^γ+^Α^γ)/α. Data are plotted as mean ± s.d. (*n* = 2). **(D)** Quantification of immunoblots in a chart showing the % of (^G^γ+^Α^γ)/α expression in each target on days 4 and 9 of erythroid differentiation. Data are plotted as mean ± s.d. (*n* = 2).

After confirmation of efficient editing and creation of the required mutations, the edited HUDEP-2 cells were subjected to erythroid differentiation. On days 4 and 9 of differentiation, cells were collected for functional assays at the protein level, including immunoblot and HPLC analyses. HPLC analysis on day 9 of differentiation allowed quantification of γ-globin induction after editing. The HPLC chromatograms indicated γ-globin induction with the *BCL11A* and *HBG* targets compared to mock samples (Figure 3A; Supplementary Figure S2A), up from 0% γ/α relative to β/α mock levels, to 8.7% (n = 3) and 11.0% (*n* = 1) of γ/α relative to β/α mock levels, respectively (Figure 3C; Supplementary Figure S2B). Despite effective on-target editing of *KLF1* and description of the specific mutation in the context of potentially β-thalassemia-ameliorating variants in the Chinese population (Liu et al., 2014), there was no indication of γ-globin induction in a single assessment (*n* = 1), indicating that the mutation in question might not be γ-globin-inducing after all (Supplementary Figure S2), at least in the widely used HUDEP-2 cell line of Japanese origin (Cell Engineering Division - Cell Bank, 2013). These results for relative γ-globin levels after treatment were validated in parallel to HPLC analyses by immunoblots after collection of cell material on days 4 and 9 of erythroid differentiation (Figures 3B, D; Supplementary Figures S2C, D). Quantification of γ-globin levels in edited samples indicated up to 50% (day 9) and 70% (day 4) γ/α levels in *BCL11A*- and *HBG*-edited cells, respectively, compared to 0% γ/α in both mock- and *KLF1*-targeted cells for either time point.

### 3.3 Delivery of TALENs as mRNA for β-globin induction in hematopoietic cells

**FIGURE 4:**
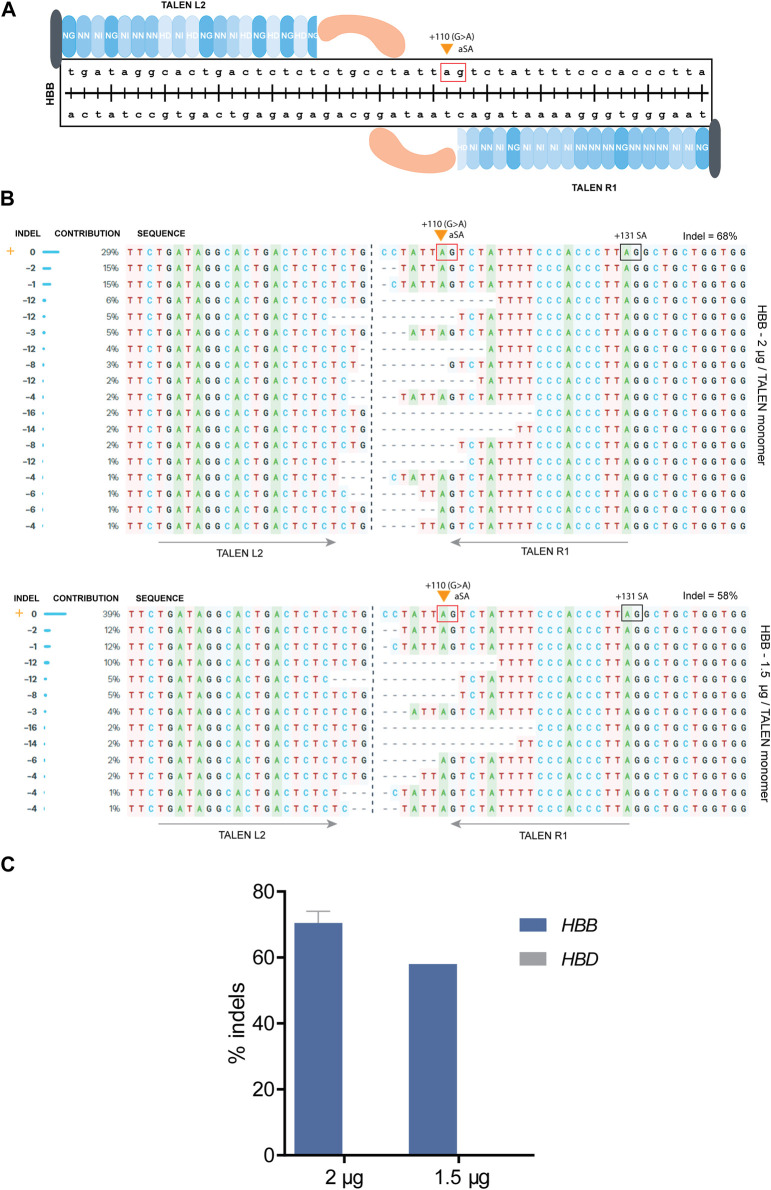
TALEN-based targeting of the *HBB* ^*IVSI−110(G>A)*^ mutation in patient-derived CD34^+^ cells. **(A)** A schematic diagram showing the design of TALENs targeting the *HBB* ^*IVSI−110(G>A)*^ mutation (orange arrow). The red box shows the aSA (AG dinucleotide) that is created by the mutation. The TALEN monomers L2 and R1 were used to introduce DSBs and create indels upstream of the splice acceptor site. The blue shaded repeated shapes represent the specific RVDs used for the design, the black shape shows the N-terminus, while the orange shape represents the Fok1 endonuclease monomer. **(B)** Alignment of the various editing events resulting from the disruption of the upstream splice acceptor site using the TALENs R1/L2 pair. For the top alignment 2 μg*,* for the bottom 1.5 μg were used per TALEN monomer. The INDEL column shows the exact number of deleted (<0) nucleotides, the CONTRIBUTION column the percentage of these indels in the bulk population*,* and the SEQUENCE column the edited sequences containing the various indels aligned with the wild type (orange cross). The orange arrow indicates the *HBB* ^*IVSI−110(G>A)*^ mutation as the first base of the aSA (red box); the normal +131 splice acceptor site (SA) is indicated by a black box. The grey arrows show the binding site of each TALEN monomer. The percentage of the total indels is also displayed. **(C)** Chart showing the percentage of indels detected for *HBB* and *HBD* after delivery of either 2 or 1.5 μg per TALEN monomer. For the 2 μg/TALEN monomer*,* the data are plotted as mean ± s.d. (*n* = 2.)

A different approach for therapy of β-hemoglobinopathies is the elevation of β-globin production by correcting the causative mutation, which for some mutations can be achieved by simple NHEJ-based disruption based on a single DSB. For instance, the *HBB* ^*IVSI−110(G>A)*^ mutation creates an aberrant splice acceptor (aSA) site that leads to abnormal splicing, which according to previous studies of our group can be effectively restored to normal by CRISPR- or TALEN-mediated disruption of aberrant regulatory elements required for abnormal splicing (Patsali et al., 2019a; Patsali et al., 2019b). Specifically, for TALENs, the TALEN pair R1/L2 was designed to target the *HBB* gene with a predicted DSB just upstream of the *HBB* ^*IVSI−110(G>A)*^ mutation and the aSA consensus sequence (i.e., GA, Figure 4A). The results suggested that deletion of nucleotides upstream of the mutation can lead to correction of HBB expression even when the aSA remains intact (Patsali et al., 2019a; Patsali et al., 2019b), e.g., when TALEN-mediated sequence deletions shorten the aSA distance to the upstream branchpoint site sufficiently in order to destabilize lariat formation for aSA-based splicing and to favor normal splicing instead.

Toward clinical translation of the approach, long-term safety and efficiency analyses are required. In the process of optimizing genome editing efficiency, different amounts of TALEN mRNA (2 and 1.5 μg per TALEN monomer) were evaluated for *in vitro* and *ex vivo* delivery. Here, we show that based on the current *in vitro* transcription protocol for the production of TALEN mRNA, high editing efficiencies can be achieved by nucleofection of *HBB* ^*IVSI−110(G>A)*^ homozygote human CD34^+^ cells, which can lead to the correction of aberrant splicing and improvement of disease parameters, such as the restoration of HBB expression, correction of *HBB* pre-mRNA splicing and advanced erythroid differentiation. A key concern for editing of globins is the specificity of editing tools, owing to the high similarity of *HBB* and *HBD* (Liang et al., 2015), which can lead to unproductive *HBD*-*HBB* gene fusions and even overall reduced HBB expression (Patsali et al., 2019b). We therefore investigated the effect of different mRNA amounts on both, on-target editing efficiency and the ratio of on- to off-target editing.

Patient-derived CD34^+^ cells were nucleofected, providing the TALEN R1/L2 nuclease as mRNA at a 1:1 ratio, either using 2 or 1.5 μg per monomer, followed by culture and analysis of mixed sequence traces by ICE (Conant et al., 2022) to derive editing efficiency and sequence information for the treated cell population. After nucleofection with 2 μg mRNA/TALEN monomer, up to 17 different deletions were observed, in the absence of insertions. Of those deletions, 9 removing the *HBB* ^*IVSI−110(G>A)*^ mutation contributed 39.7% to all indel events, while the remaining 8 deletions occurred upstream of the mutation with up to 60.3% contribution to all indel events. The greatest contribution to indel events was made by short, 1–2 base pair (bp) deletions (44%). In total, short deletions of <10 bp accounted for 67.7% of indels, while only 32.3% of indel events were contributed by deletions >10 bp. After nucleofection with 1.5 μg mRNA/TALEN monomer, up to 12 different deletions were detected, once more in the absence of insertions. 5 out of those 12 deletions were targeting the *HBB* ^*IVSI−110(G>A)*^ mutation directly and contributed 37% to all indel events, while the remaining 7 deletions were located upstream of the mutation with 58.6% contribution to all indel events. The commonest indel events were 1–2 bp deletions (41.4%). 67.2% of the events were <10 bp deletions, while, 32.8% were >10 bp deletions (Figure 4B). Most importantly, while the distribution of indel events was similar for both conditions, the final editing efficiency score was 70.5% with 2 μg and 58% with 1.5 μg (Figure 4C). Moreover, there was no detectable editing of *HBD* for either input amount*,* indicating minimal *HBD* off-target activity and minimal scope for inadvertent fusion events. The results indicated that further escalation of mRNA amounts may raise editing efficiency still more, without prompting inadvertent *HBD* edits.

## 4 Discussion

Genome editing of hematopoietic cells has already reached significant milestones. For inherited disorders this includes early achievement of treatment end points by CTX001 treatment of β-hemoglobinopathies in clinical trials (Frangoul et al., 2020) and many other preclinical and clinical evaluations (Koniali et al., 2021; Cleared et al., 2022), and for cancer treatment this includes clinical application of autologous CAR-T cells and allogeneic CAR-NK and CAR-T cells (Ottaviano et al., 2022; Valeri et al., 2022; Zhang et al., 2022). Much of this progress is based on *ex vivo* delivery by electroporation, which when suitably optimized achieves great efficiencies even in HSPCs as cells recalcitrant to transfection (Hoban et al., 2015; Frangoul et al., 2021; Chu et al., 2021). In clinically relevant cells, DNA delivery is marked by low editing efficiencies and high toxicity, whereas protein- or RNP-based delivery allows high efficiencies but is cost- and labor-intensive. This is particularly true when new targets are explored by protein-based recognition typical of ZFNs and TALENs (in contrast to what applies for gRNA-mediated recognition for CRISPR/Cas), which requires dedicated protein synthesis for each new target (Gaj et al., 2015). By contrast, mRNA- or mRNA/gRNA-based delivery offer near-universal applicability, high speed of adoption and application, low toxicity and high editing efficiencies. For the example of hemoglobinopathies, a growing body of work demonstrates that delivery of *in vitro* transcribed genome editing tools, such as BEs and TALENs in sickle-cell-disease and β-thalassemic cells, achieved high levels of functional correction and reached high editing efficiencies (40%–80%) (Patsali et al., 2019b; Gaudelli et al., 2020; Newby et al., 2021; Antoniou et al., 2022). Depending on the setup, mRNA-based delivery might vastly outperform editing efficiency achieved by RNPs for BEs (Newby et al., 2021), while potentially modulating viral and immune transcriptional signatures and overall reducing DNA-damage, cell-cycle and metabolic response pathways in HSPCs (Cromer et al., 2018). Both tolerability and efficiency of mRNA-based delivery can be improved by protocol and chemistry refinements, e.g. by replacement of uridine with base analogues, such as 5-methoxyuridine (Vaidyanathan et al., 2018). A high-level baseline protocol facilitates any such optimization, and to date a comprehensive description of the required reagents and procedures for application of CRISPR/Cas and TALENs in CD34^+^ and HUDEP-2 cells and of typical outcomes as a benchmark for direct adoption by others in the field has been missing. In this study, we present a detailed, simple, affordable and fast *in vitro* transcription protocol for the production of mRNAs and RNA-based delivery of BEs or TALENs and for their nucleofection in HUDEP-2 and patient-derived CD34^+^ cells, conducive to reproducibly high editing efficiencies.

In a first typical application, base editing technology was employed to target modifiers of globin expression and to achieve induction of γ-globin. In the process we used the BE4max plasmid as template for the *in vitro* transcription protocol. Confirmation of BE mRNA production was followed by nucleofection of HUDEP-2 cells with three different gRNA/BE mRNA combinations, respectively targeting the +58 *BCL11A* erythroid enhancer, the *HBG* promoter, and exon 1 of the *KLF1*. Based on deconvolution of mixed sequencing traces, high editing efficiencies were achieved ranging from 30%–80%, with the highest on-target editing observed in *BCL11A* and *KLF1*, and the lowest in *HBG*. Low DNA editing efficiencies for the *HBG* promoter agree with findings in other studies indicating that the target itself rather than vectors or delivery method may be suboptimal for editing (Zhang et al., 2020), vindicated here by much higher editing efficiencies for other targets based on commercial gRNAs and same-batch application of BE mRNA. Overall, our protocol for *in vitro* transcribed BE mRNA allowed high editing efficiencies after nucleofection of HUDEP-2 cells, comparable to same-target RNP transfection of Cas9 nucleases or BEs in HSPCs (Wu et al., 2019). Importantly, Zeng *et al*, suggested that two rounds of electroporation were required to achieve high editing efficiencies by using the A3A (N57Q)-BE3 editor as RNP complex with a sgRNA targeting the +58 *BCL11A* erythroid enhancer (Zeng et al., 2020). However, in this study and in a recent study by Antoniou *et al*, it is suggested that one mRNA-based nucleofection of BEs is adequate to reach high efficiencies in HSPCs (Antoniou et al., 2022). Furthermore, deconvolution of sequence traces did not show any edits outside the predicted editing window (position 4–8 of sgRNA) for *BCL11A* and *HBG* as targets, and only the intended transition events (C>T) and no transversion events (C>A, C>G) were detected above background. For *KLF1* as target, one edit outside the standard editing window was detected, for the C at position 3 of sgRNA, which is consistent with previous observations for BE4max (Koblan et al., 2018). Koblan *et al.* had observed C>T transition edits at high efficiency for positions 4–8 of the protospacer, but also at moderate to low efficiency at positions 3, 9, and 12 (Koblan et al., 2018). In particular for application of BEs in open reading frames, bystander edits including those outside the editing window need to be borne in mind. Luckily for the presented target and application, the single detectable bystander edit represents a synonymous mutation compared to the intended amino acid substitution of glutamic acid to lysine and still allowed the evaluation of potential effects of the protein variant on HbF expression. To this end we employed erythroid differentiation and analyses by RP-HPLC and immunoblots after confirming high editing efficiencies. Both methods revealed presence of γ-globin induction for the *BCL11A* and *HBG* targets and its absence for the *KLF1* target. As to the former, our results agree with other studies showing increases of γ-globin for the specific *BCL11A* and *HBG* targets (Zeng et al., 2020; Zhang et al., 2020) and indicating their relevance for therapeutic application of genome editing. As to the latter, it had been reported by Liu *et al.* that mutations in *KLF1*, such as p.Glu5Lys C>T, can ameliorate the severity of β-thalassemia by contributing to increased HbF levels (Liu et al., 2014). However, at high editing efficiencies made possible by the present protocol, our preliminary analysis indicates that the specific *KLF1* variant has no detectable effect on γ-globin levels compared to negative controls and may therefore have little therapeutic relevance for β-hemoglobinopathies.

In a second typical application, TALEN tools were exploited for the correction in patient-derived CD34^+^ cells of a pathogenic single-nucleotide variant, the common *HBB* ^*IVSI−110(G>A)*^ mutation. The cells were nucleofected with two different mRNA quantities, 2 and 1.5 μg, per TALEN monomer, targeting the upstream region of the +110 G>A mutation located in *HBB* intron 1. Seven days post-nucleofection, cells were collected for sequence analysis and showed high editing efficiencies up to 70% with 2 μg per TALEN monomer and up to 58% with 1.5 μg per TALEN monomer. For our own experiments, this indicated 2 μg per TALEN monomer as preferable for efficient editing by nucleofection. Results obtained here rival the highest efficiencies reported elsewhere (Patsali et al., 2019b; Lux et al., 2019; Quintana-Bustamante et al., 2019), indicating the success of our protocol to utilize *in vitro* transcribed TALEN mRNAs for nucleofection and editing in HSPCs. In line with other TALEN studies, targets and delivery methodologies (Kim et al., 2013; Ma et al., 2014; Wang et al., 2015; Patsali et al., 2019a; 2019b), we found that NHEJ only resulted in deletions and no insertions in the present case. Importantly, it has been reported elsewhere that because of the high sequence similarity between *HBB* and *HBD* genes, targeting the *HBB* gene resulted in high off-target indels in *HBD* (Cottle et al., 2015; Liang et al., 2015). Here, we confirm reduction below the detection limit of off-target editing for the *HBD* gene with the spacer-optimized TALEN L2/R1 combination (Patsali et al., 2019a; Patsali et al., 2019b). This would also keep any recombination events between both loci to a minimum, although this has not been assessed directly in this study, e.g., by CAST-seq (Turchiano et al., 2021). In addition to high on-target precision, we also demonstrated the integrity of the proximal *HBB* exon 2 and its consensus splice acceptor site for all inferred editing events, altogether vindicating the suitability of TALEN L2/R1 for therapeutic application.

In conclusion, the present manuscript provides a comprehensive, fast and readily reproducible protocol for the production and application of genome editing tools as mRNAs, based on an *in vitro* transcription process and the nucleofection of these products in hematopoietic cells. The transfection of BEs and TALENs as mRNA in otherwise hard-to-transfect cells leads to high editing efficiencies that facilitate functional analyses for the investigation of potential therapies for β-hemoglobinopathies and beyond.

## Data Availability

The raw data supporting the conclusion of this article will be made available by the authors, without undue reservation.
