# Supplementary material for: High-efficiency editing in hematopoietic stem cells and the HUDEP-2 cell line based on in vitro mRNA synthesis
Source: Front Genome Ed. 2023 Mar 8;5:1141618. doi: 10.3389/fgeed.2023.1141618 (PMC10030607; doi:10.3389/fgeed.2023.1141618)
Supplement: Supplementary file 1 [file DataSheet1.PDF]

Supplementary Material

1 Supplementary Tables

Supplementary Table 1. List of base editors, their gRNAs, PAM sequence, editing window (with targeted bases shown in bold red font) and the targeted gene. All sequences are given 5’ to 3’ on the target site.

Base editor	gRNA	PAM	Editing window	Gene
pCMV_BE4max	TTTATCACAGGCTCCAGGAA	GGG	AT**C**A**C**	*BCL11A*
pCMV_BE4max	CTTGACCAATAGCCTTGACA	AGG	GA**CC**A	*HBG (-114 C>T / -115 C>T)*
pCMV_BE4max	GTCTCGGCTGTGGCCATGGC	TGG	T**C**GG**C**	*KLF1 (p.Glu5Lys)*

Supplementary Table 2. List of TALEN monomer RVDs and binding sequences. The RVD sequences are given from the N-terminus to the C-terminus and binding sequences 5’ to 3’.

Monomer name	RVD sequence	Binding sequence
TALEN R1	NG NI NI NN NN NN NG NN NN NN NI NI NI NI NG NI NN NI HD	TAAGGGTGGGAAAATAGAC
TALEN L2	NG NN NI NG NI NN NN HD NI HD NG NN NI HD NG HD NG HD NG	TGATAGGCACTGACTCTCT

Supplementary Table 3. List of all primers used for Sanger sequencing. For each primer pair (forward / reverse) the target locus, sequence, annealing temperature (T_A) and product size (Amplicon) are given.

Primer Pair	Target locus	Sequence (5’–3’)	T_A (°C)	Amplicon (bp)
BCL11A_Enh_FW / BCL11A_Enh_R	*BCL11A*	ACCCTGGAAAACAGCCTGAC / GAGAGTGCAGACAGGGGAAG	68	578
HBG_prom_FW / HBG_prom_RV	*HBG*	ATCGGAACAAGGCAAAGGCTA / GGCGTCTGGACTAGGAGCTT	68	307
KLF1_exon1_FW / KLF1_exon1_RV	*KLF1*	CTTTGGACACAGGGTTAGTC / GTACCTCAGTCCTGGTTAAG	64	436
HBB_FW / HBB_RV	*HBB*	TGAGGAGAAGTCTGCCGTTAC / CAGCTCACTCAGTGTGGC	66	389
HBD_FW / HBD_RV	*HBD*	TGAGGAGAAGACTGCTGTCAA / CAGTGCAGCTCACTCAGCT	66	392

Supplementary Table 4. Primary and secondary antibodies used in immunoblotting; BSA – bovine serum albumin, TBST – 1× Tris-Buffered Saline, 0.1% Tween 20 Detergent, SMP – skimmed milk powder

Type	Description and catalog number	Dilution	Diluent
**Primary antibodies**	Hemoglobin γ (51-7) mouse monoclonal IgG1; sc-21756	1:1000	1x BSA/TBST
	Mouse monoclonal anti-human HBB (37-8); sc-21757		
	Haemoglobin α (H-80) rabbit polyclonal IgG; sc-21005		
	Anti-β-actin (AC-15) mouse monoclonal IgG1; A-1978	1:10000	1% SMP/TBST
**Secondary antibodies**	Peroxidase-conjugated AffiniPure goat anti-mouse IgG (H+L); 115-035-003	1:8000	3% SMP/TBST
	Peroxidase-conjugated AffiniPure goat anti-rabbit IgG (H+L); 111-035-003		

2 Supplementary Figures

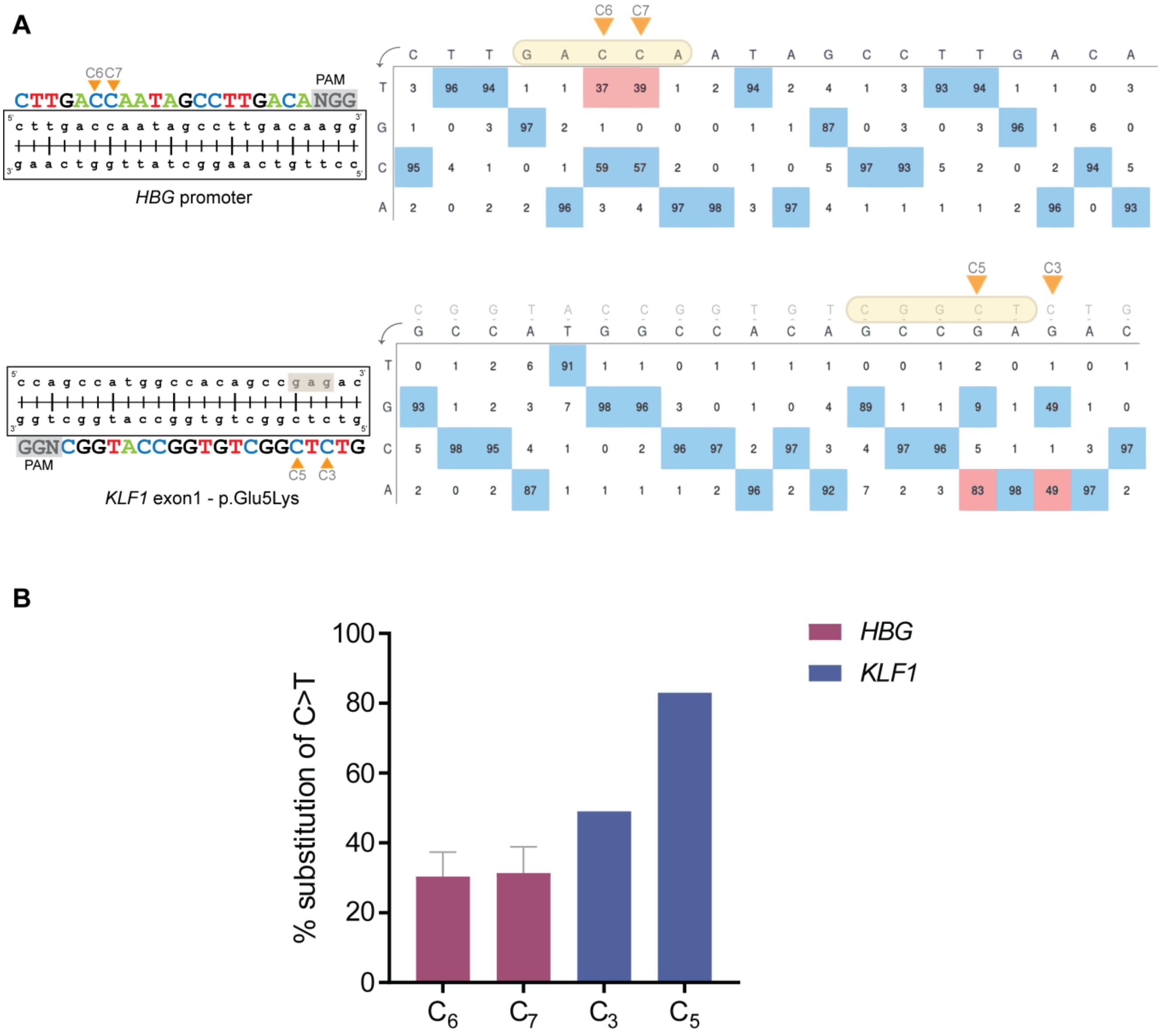

Supplementary Figure 1. Editing efficiency with BE in HUDEP-2 cells. (**A**) (left) Schematic diagram illustrating the *HBG* and *KLF1* targets with the DNA-equivalent of the 20-nt gRNA and the 3-nt PAM sequence. The orange arrows show the C bases that were edited. The highlighted rectangle indicates the GAG codon (Glutamine acid) at *KLF1* target. (right) Annotated EditR-generated plot illustrating the percent area of the signal for each base (A|C|G|T) at the corresponding gRNA position for edited HUDEP-2 cells. The highlighted shape shows the editing window, the orange arrows show the edited C bases and the red boxes display the exact percentage (%) of base substitution in the bulk cell population. (**B**) Chart showing the % base substitution of C>T after base editing. Each bar shows the editing of the corresponding C base along the gRNA with different bar color for each target.

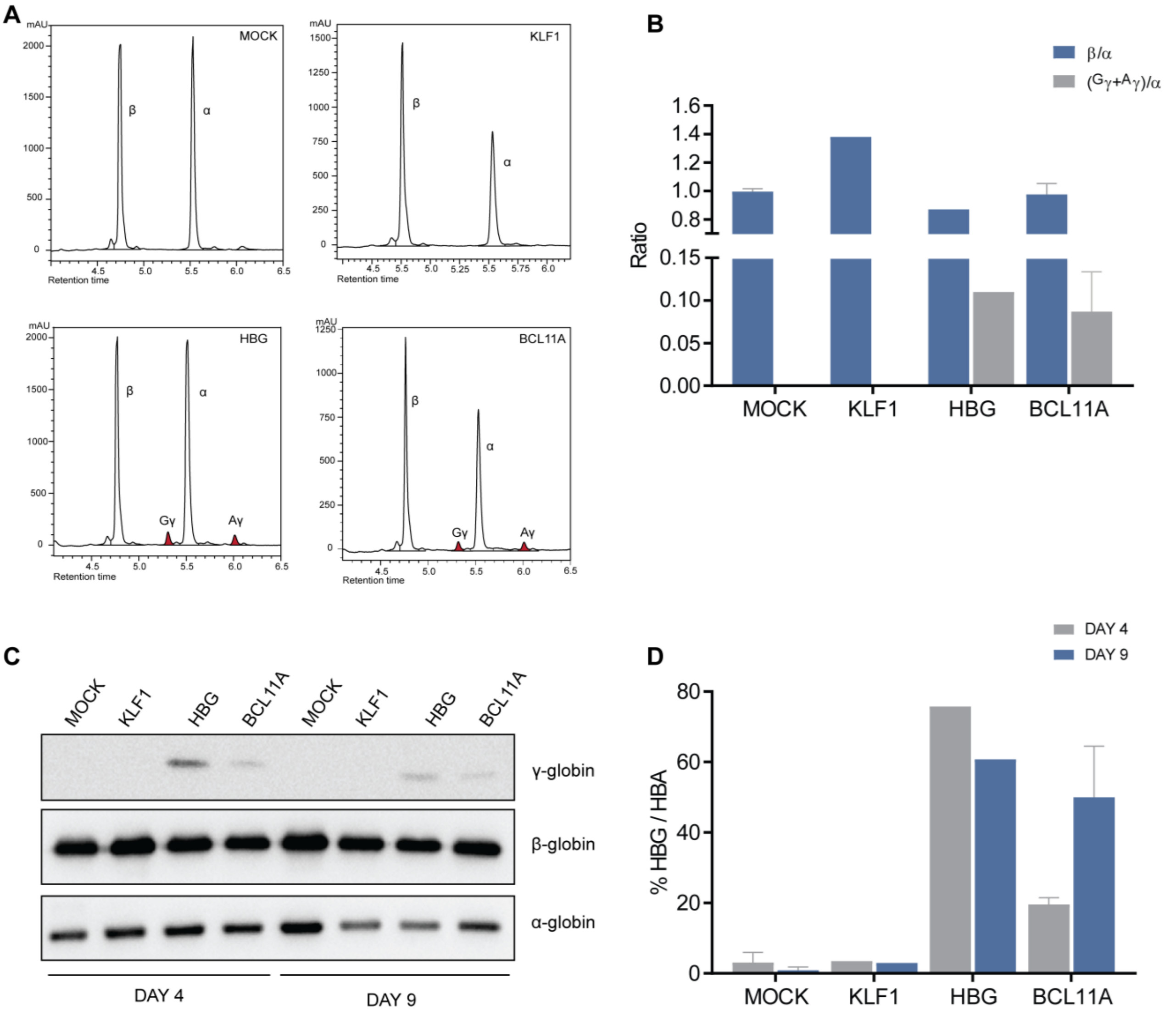

Supplementary Figure 2. Functional analysis of BE-modified HUDEP-2 cells. (**A**) Chromatograms of HPLC analysis for the *KLF1*, *HBG* and *BCL11A* targets and mock at the last day of HUDEP-2 erythroid differentiation, with the peaks showing globin expression. The α-, β- and γ-globin peaks are labelled, and colored peaks indicate γ-globin induction. (**B**) Quantification of HPLC analysis in a chart showing the globin ratios of β/a and ($^{G}\gamma$+$^{A}\gamma$)/α (**C**) Immunoblots of the edited HUDEP-2 cells on days 4 and 9 of erythroid differentiation, detecting the protein expression of α-, β- and γ-globin. Here, the whole image of immunoblots is presented including all three targets (*BCL11A*, *HBG* and *KLF1*), while in figure 3 (Results section) the immunoblot images were spliced to emphasize the BCL11A target. (**D**) Quantification of immunoblots in a chart showing the percentage of ($^{G}\gamma$+$^{A}\gamma$)/α expression in each target on days 4 and 9 of erythroid differentiation.
